# Involved‐Field Irradiation Versus Elective Nodal Irradiation in Patients With Locally Advanced Esophageal Squamous Cell Carcinoma Treated With Neoadjuvant Chemoradiotherapy

**DOI:** 10.1002/cam4.71392

**Published:** 2025-11-30

**Authors:** Xiaoding Zhou, Ying Liu, Jie Zhu, Jingqiu Li, Yi Wang, Guiyu Huang, Lin Peng, Yongtao Han, Xuefeng Leng, Chenghao Wang, Wenwu He, Lei Wu, Qifeng Wang

**Affiliations:** ^1^ Department of Radiation Oncology, Radiation Oncology Key Laboratory of Sichuan Province, Sichuan Clinical Research Center for Cancer, Sichuan Cancer Hospital & Institute, Sichuan Cancer Center University of Electronic Science and Technology of China Chengdu China; ^2^ Department of Thoracic Surgery, Sichuan Cancer Hospital & Institute, Sichuan Cancer Center, School of Medicine Chengdu China

**Keywords:** elective nodal irradiation, involved‐field irradiation, locally advanced esophageal squamous cell carcinoma, neoadjuvant chemoradiotherapy, out‐of‐field lymph nodes

## Abstract

**Background:**

The method of lymph node (LN) irradiation for locally advanced esophageal squamous cell carcinoma (LA‐ESCC) is still a topic of debate. We investigated the efficacy, toxicity, and rate of out‐of‐field LNs in irradiation across different target areas in patients with LA‐ESCC undergoing neoadjuvant chemoradiotherapy (nCRT).

**Methods:**

We retrospectively reviewed patient records from June 2017 to August 2022 and divided patients into elective nodal irradiation (ENI) and involved‐field irradiation (IFI) groups. The differences in hematological and non‐hematological toxicities of the out‐of‐field LNs were analyzed between the two groups. The log‐rank test was used to evaluate the Kaplan–Meier curves for overall and progression‐free survival.

**Results:**

Among the 306 included patients, 202 (66.0%) received ENI and 104 (34.0%) received IFI. At the 3‐year follow‐up, the survival rate did not differ significantly between the groups (*p* > 0.05). Although the occurrence of radiation‐induced pneumonia did not differ (*p* > 0.05), the incidence of radiation‐induced esophagitis and the degree of leukopenia differed significantly (*p* < 0.05). While the average heart irradiation dose or heart V_20_, V_30_, and V_40_ did not differ significantly (*p* > 0.05), we observed significant differences in the clinical target volume, average lung irradiation dose, and lung V_20_, V_30_, and V_40_ (*p* < 0.05). Among all patients, 29 cases (9.5%) experienced out‐of‐field LNs with 26 (93.1%) in abdominal LNs, whereas only 3 cases (6.9%) with out‐of‐field LNs were in the upper esophagus. There was no statistical significance between out‐of‐field LNs and LN irradiation methods (*p* = 0.724).

**Conclusions:**

Under similar prognostic conditions, IFI resulted in mild toxicity compared to ENI. Therefore, for patients with ESCC undergoing nCRT, IFI is the preferred irradiation approach for the lymphatic drainage area.

## Abstract 2

Our study boasts the largest sample size among existing research on ESCC patients who have undergone nCRT treatment. For LA‐ESCC patients who have undergone nCRT, IFI is the preferred method of lymph node irradiation.

**Abbreviations**
BMI: body mass index
CI: confidence intervals
CT: computed tomography
CTV: clinical target volume
DVH: dose‐volume histograms
ECOG: Eastern Cooperative Oncology Group
ENI: elective nodal irradiation
ESCC: esophageal squamous cell carcinoma
GTV: gross tumor volume
HR: hazard ratio
IFI: involved‐field irradiation
KPS: Karnofsky performance status
LA‐ESCC: locally advanced esophageal squamous cell carcinoma
LNs: lymph nodes
nCRT: neoadjuvant chemoradiotherapy
OS: overall survival
pCR: pathological complete response
PET‐CT: positron emission tomography‐computed tomography
PFS: progression‐free survival
ROI: region of interest
RTOG: Radiation Therapy Oncology Group

## 1 Background

Esophageal cancer is a widespread cancer, ranking sixth among all cancer‐related deaths worldwide [1]. Esophageal squamous cell carcinoma (ESCC) is the primary cause of esophageal cancer and accounts for 85% of all cases [2]. The typical treatment for locally advanced ESCC (LA‐ESCC) combines neoadjuvant chemoradiotherapy (nCRT) with radical esophagectomy [3, 4, 5, 6]. Patients who receive nCRT have a median survival rate that is four times greater than that of patients who undergo surgery alone [3].

LA‐ESCC frequently metastasizes to the lymph nodes (LNs) [7]; however, the best radiotherapy approach for LN drainage zones is still a topic of debate [8]. An optimal irradiation range aims to strike a well‐balanced equilibrium between the adverse effects and toxicities associated with radiation therapy (such as radiation esophagitis, radiation pneumonitis, and leukopenia) and the potential survival benefits; it seeks to maximize treatment outcomes for patients while reducing the chances of complications arising from radiotherapy. Elective nodal irradiation (ENI) was previously the most popular method for outlining LN drainage areas in patients with esophageal cancer; however, this approach can lead to severe radiation side effects [9]. Currently, involved‐field irradiation (IFI) is increasingly employed by radiation oncologists [10]. Evidence has demonstrated that ENI and IFI do not affect overall survival (OS) or progression‐free survival (PFS) in patients with ESCC undergoing nCRT [11, 12, 13, 14]. A meta‐analysis covering 23 studies with 4120 patients revealed no significant increase in OS rates or instances of Grade ≥ 3 acute pneumonia in the IFI group; however, a significant decrease in Grade ≥ 2 acute esophagitis cases was reported [13]. On the contrary, other researchers have reported a longer median OS for ENI compared to that for IFI [15]. Therefore, there remains no agreement on the two LN irradiation strategies. In addition, the rate of out‐of‐field LN irradiation and areas with high levels of out‐of‐field LNs have not been studied extensively, particularly because of the dearth of large‐scale cohort studies on nCRT followed by radical esophagectomy.

Thus, we conducted a retrospective study to investigate the efficacy, toxicity, and target areas of IFI and ENI in patients with ESCC undergoing nCRT. Additionally, we compared the rate of out‐of‐field LNs between the two groups.

## 2 Methods

### 2.1 Patients and Datasets

We conducted a retrospective analysis of patient data from Sichuan Cancer Hospital & Institute in Chengdu, China, focusing on those who underwent curative‐intent esophagectomy between June 2017 and August 2022. Our inclusion criteria comprised the following: (1) patients within the age range of 18–80 years; (2) histological confirmation of stage II–IVA ESCC based on the 8th American Joint Committee on Cancer TNM staging system [16]; (3) a performance status of 0–1 as defined by the Eastern Cooperative Oncology Group (ECOG) [17]; (4) treatment with standard nCRT with radical esophagectomy; and (5) complete radiotherapy data and imaging dose‐volume histograms (DVHs). The exclusion criteria were as follows: (1) double primary cancer; (2) pathologically verified non‐squamous cell carcinoma; (3) palliative esophagectomy; (4) simple surgical treatment or radical radiotherapy; and (5) missing computed tomography (CT) or clinical target volume (CTV) data. This retrospective study was performed in accordance with the principles of the Declaration of Helsinki and approved by the Ethics Committee of Sichuan Cancer Hospital, which waived the requirement for written informed consent (ethics number, SCCHEC‐02‐2023‐029).

### 2.2 Treatment Methods

#### 2.2.1 Radiotherapy

Gross tumor volume (GTV) was delineated based on clinical imaging assessments including esophagoscopy, CT, and positron emission tomography‐computed tomography (PET‐CT). The use of endoscopic ultrasound examination allowed accurate staging. Two radiation oncologists with > 10 years of experience co‐developed the radiation treatment plans. The radiation doses ranged from 30.0 to 50.0 Gy, with a fraction size of 1.8 to 2.0 Gy delivered once daily, five times per week. The tumor was delineated as the region of interest (ROI) via MIM 5.6 (MIM Software Inc.).

##### 2.2.1.1 ENI

The GTV was defined as the area with esophageal wall thickening ≥ 0.5 cm on CT scan or the exclusion of regions with a maximum luminal diameter of the gas‐filled esophagus of ≥ 1 cm, while considering the results of esophageal barium meal imaging and endoscopic examination for adjustments. The CTV was delineated by enlarging the GTV 0.5–0.8 cm laterally and 2–3 cm vertically, adjusted for anatomical boundaries and metastatic LN positions. The selective lymphatic drainage area was referred to as ENI (including the bilateral supraclavicular, paraoesophageal, 2nd, 4th, 5th, and 7th zones and a 3–4 cm range below the carina).

##### 2.2.1.2 IFI

The IFI irradiation scheme for ESCC, the metastatic LN delineation method, and CTV expansion methods were the same as those in the ENI group. The definition of IFI is limited to involved LNs and does not consider the LN drainage area. The criteria for determining metastatic LNs were a short‐axis diameter of > 0.5 cm for paratracheal and paraoesophageal LNs and a short‐axis diameter of > 1 cm for other LNs.

#### 2.2.2 Chemotherapy and Surgery

The patients received concurrent chemotherapy and radiotherapy. The main chemotherapy regimen consisted primarily of paclitaxel (135–175 mg/m^2^, day 1) and carboplatin (cisplatin), with a total of 1–4 cycles (often two cycles) administered. Each treatment cycle was 3 weeks. Surgery took place 4–8 weeks following the end of nCRT. The surgical approach encompassed Ivor Lewis or McKeown esophagectomy with a three‐field LN removal.

### 2.3 Follow‐Up

Follow‐up evaluations were performed at regular intervals every 6 months during the first 2 years and annually thereafter. The efficacy of the treatment was gauged based on the Response Evaluation Criteria in Solid Tumors (version 1.1) [18]. Dates of death were acquired from clinical records, telephone calls to relatives of the deceased, or from the central registry of the Chinese Bureau of Population Statistics.

### 2.4 Evaluation Indicators

(1) OS was defined as the time from treatment initiation until death from any cause. PFS was defined as the time from treatment initiation to disease progression. Pathological complete response (pCR) was defined as the absence of gross or microscopic tumor tissue in both the primary lesion and LNs upon examination of the surgical specimen. (2) Radiation‐related toxicities were compared between the two groups of patients according to the Radiation Therapy Oncology Group (RTOG) grading toxicity criteria [19] and included radiation esophagitis, acute pneumonitis, neutrophil count decrease, hemoglobin and platelet decrease. (3) Dose and volume assessment of target organs and CTV (e.g., “V_20_ of lung” refers to the percentage of the total lung volume receiving a dose of 20 Gy or higher). (4) Out‐of‐field LN rates (referring to positive lymph nodes that were not included in the radiotherapy target area).

### 2.5 Statistical Analysis

R language (version 4.0.3) and IBM SPSS Statistics for Windows, version 26.0, were used to perform the statistical analyses. The baseline characteristics of the two treatment groups and toxicity were compared using the chi‐squared test. The *t*‐test was used to compare dosimetry differences. We used the log‐rank test to evaluate the Kaplan–Meier curves for OS and PFS. Multivariate Cox analyses were performed for variables with *p* < 0.05 in univariate analysis. Results of the Cox regression analysis and K‐M curve were summarized as hazard ratio (HR), 95% confidence intervals (CIs), and P value. A *p*‐value < 0.05 denoted a significant difference between the groups (two‐sided tests). A subgroup analysis was performed specifically for patients older than 70 years.

## 3 Results

### 3.1 Patient Characteristics

**FIGURE 1:**
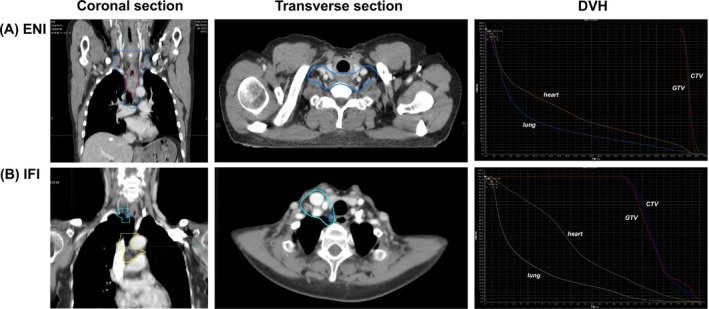
Example diagram of the lymph node irradiation method. Irradiation field of neoadjuvant radiotherapy using ENI (A) and IFI (B) for two patients with upper thoracic esophageal carcinoma, including coronal and transverse sections of computed tomography and dose and volume histograms (DVH). ENI, elective lymph node irradiation; IFI, involved‐field irradiation.

Between June 2017 and August 2022, among the 391 patients who were diagnosed with ESCC and underwent nCRT at our center, 306 were ultimately included in our analyses. The inclusion and exclusion information is shown in Figure S1. ENI was administered to 202 patients and IFI was administered to 104 (Figure 1). In the ENI group, 33.2% of the patients achieved pCR, whereas in the IFI group, 27.9% achieved pCR; there was no statistically significant difference between the two groups (*p* = 0.27). The median follow‐up period for all patients was 31.8 months (range, 9.6–64.0 months). The baseline characteristics of the patients in the two groups are presented in Table 1. Sex, age, body mass index (BMI), ECOG, performance status, and Karnofsky performance status (KPS) did not differ significantly (*p* > 0.05). There was no significant difference in the distribution of clinical N stages or the number of positive LNs at diagnosis. Cox multivariate analysis revealed no significant association (*p* > 0.05) between smoking, drinking, location, clinical stage and survival or toxicity, confirming their status as confounding variables (Tables S3–, S5).

**TABLE 1:** Patients' clinical characteristics.

Variables	ENI (*n* = 202)	IFI (*n* = 104)	*p*
Age			0.122
< 65	142 (70.3)	64 (61.5)	
≥ 65	60 (29.7)	40 (38.5)	
Sex			0.131
Male	173 (85.6)	82 (78.8)	
Female	29 (14.4)	22 (21.2)	
Smoking			0.019
No	62 (30.7)	46 (44.2)	
Yes	140 (69.3)	58 (55.8)	
Drinking			0.036
No	63 (31.2)	45 (43.3)	
Yes	139 (68.8)	59 (56.7)	
BMI			0.73
< 18.5	16 (7.9)	6 (6.7)	
18.5–24	122 (60.4)	62 (59.6)	
> 24	64 (31.7)	36 (34.6)	
ECOG			0.13
0	168 (83.2)	79 (76)	
1	34 (16.8)	25 (24)	
KPS			0.31
70–80	32 (15.8)	12 (11.5)	
90–100	170 (84.2)	92 (88.5)	
Location			< 0.001
Upside	30 (14.9)	10 (9.6)	
Middle	84 (41.6)	45 (43.3)	
Lower	84 (41.6)	33 (31.7)	
N stage			0.26
N0	11 (3.6)	6 (3)	5 (4.8)
N1	101 (33.0)	72 (35.6)	29 (27.9)
N2	157 (51.3)	97 (48)	60 (57.7)
N3	37 (12.1)	27 (13.4)	10 (9.6)
Clinical stage			0.024
II	6 (3)	6 (5.8)	
III	139 (68.8)	82 (78.7)	
IV	53 (26.2)	14 (13.5)	
pCR			0.27
Yes	67 (33.2)	29 (27.9)	
No	134 (66.3)	73 (70.2)	

Abbreviations: BMI, body mass index; ECOG, Eastern Cooperative Oncology Group; ENI, elective lymph node irradiation; IFI, involved field irradiation; KPS, Karnofsky performance status; pCR, pathologic complete response.

### 3.2 Survival

**FIGURE 2:**
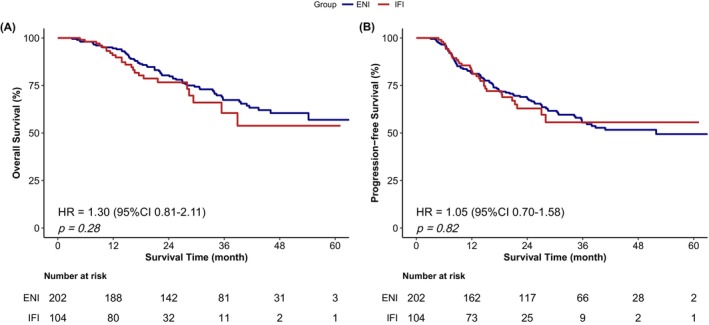
Overall survival (A) and progression‐free survival (B) for patients in the IFI group vs. ENI group. ENI, elective lymph node irradiation; IFI, involved‐field irradiation.

There was no statistically significant difference in the survival rates between the ENI and IFI groups. The median OS was not reached in our database. The respective 1‐, 3‐, and 5‐year OS rates in the ENI and IFI groups were 93.41%, 67.12%, and 57.01% versus 91.00%, 60.58%, and 54.07% (*p* = 0.28; HR = 1.30, 95% CI 0.81–2.11) (Figure 2A). The median PFS was 52.8 months in the ENI group and was not reached in the IFI group. The 1‐, 3‐, and 5‐year PFS rates were 81.37%, 55.56%, and 49.35%, respectively, in the ENI group versus 82.35%, 55.56%, and 55.56% in the IFI group (*p* = 0.82; HR = 1.05, 95% CI 0.70–1.58) (Figure 2B).

### 3.3 Toxicity

The patients were divided according to the occurrence of hematological and non‐hematological toxicities. The non‐hematological toxicity group included different levels of radiation‐induced toxicities. In this toxicity group, the incidence of acute esophagitis and radiation pneumonitis was significantly higher in the ENI group than in the IFI group (*p* < 0.001); however, the incidence of cough (*p* = 0.366), radiation mucositis (*p* = 0.055), and pulmonary infections (*p* = 0.939) did not differ significantly. In the hematological toxicity group, patients receiving IFI showed a lower degree of leukopenia (*p* < 0.001), but no significant change in platelet or hemoglobin levels (*p* > 0.05) (Table 2).

**TABLE 2:** Comparison of toxicity between ENI and IFI groups.

Variables	ENI (*n* = 202)	IFI (*n* = 104)	*p*
Radiation mucositis			0.055
0–1	194 (96)	104 (100)	
2–4	8 (4)	0 (0)	
Cough			0.366
No	154 (76.2)	84 (80.8)	
Yes	48 (23.8)	20 (19.2)	
Radiation pneumonitis			0.030
0–1	171 (84.7)	97 (93.3)	
2–4	31 (15.3)	7 (6.7)	
Pulmonary infection			0.939
0–1	187 (92.6)	97 (93.3)	
2–4	15 (7.5)	7 (6.7)	
Acute esophagitis			< 0.001
0–1	134 (66.3)	96 (92.3)	
2–4	68 (33.7)	8 (7.7)	
Leukopenia			< 0.001
0–1	74 (36.6)	62 (59.6)	
2–4	128 (63.4)	42 (40.4)	
Platelet fractionation			0.170
0–1	181 (89.6)	95 (91.3)	
2–4	21 (10.4)	9 (8.7)	
Hemoglobin fractionation			0.349
0–1	187 (92.6)	93 (89.4)	
2–4	15 (7.4)	11 (10.6)	

Abbreviations: ENI, elective lymph node irradiation; IFI, involved field irradiation.

### 3.4 Dosimetric Evaluation

The DVHs were used to calculate the dosimetry parameters. Compared with ENI, IFI notably decreased the CTV and average lung dose, along with the lung V_20_, V_30_, and V_40_ (*p* < 0.05). The average heart dose, heart V_20_, V_30_, and V_40_ showed no significant variation between groups (*p* > 0.05) (Table 3).

**TABLE 3:** Comparison of dose distribution parameters for clinical target volume and critical organs.

Variables	ENI (*n* = 202)	IFI (*n* = 104)	*p*
CTV volume (cm^3^)	282.6 ± 79.8	182.7 ± 76.4	< 0.001
Heart D_mean_ (Gy)	23.5 ± 8.4	25.0 ± 7.4	0.164
Heart V_20_ (%)	56.06 ± 24.66	61.25 ± 23.00	0.099
Heart V_30_ (%)	31.70 ± 12.97	32.40 ± 12.02	0.667
Heart V_40_ (%)	18.90 ± 8.20	18.38 ± 7.33	0.609
Lung D_mean_ (Gy)	12.9 ± 2.3	11.7 ± 2.9	< 0.001
Lung V_20_ (%)	21.80 ± 4.67	18.89 ± 6.00	< 0.001
Lung V_30_ (%)	13.27 ± 3.48	10.59 ± 4.60	< 0.001
Lung V_40_ (%)	7.91 ± 2.78	5.73 ± 3.35	< 0.001

Abbreviations: CTV, clinical target volume; ENI, elective lymph node irradiation; IFI, involved field irradiation.

### 3.5 Out‐Of‐Field Lymph Nodes

**FIGURE 3:**
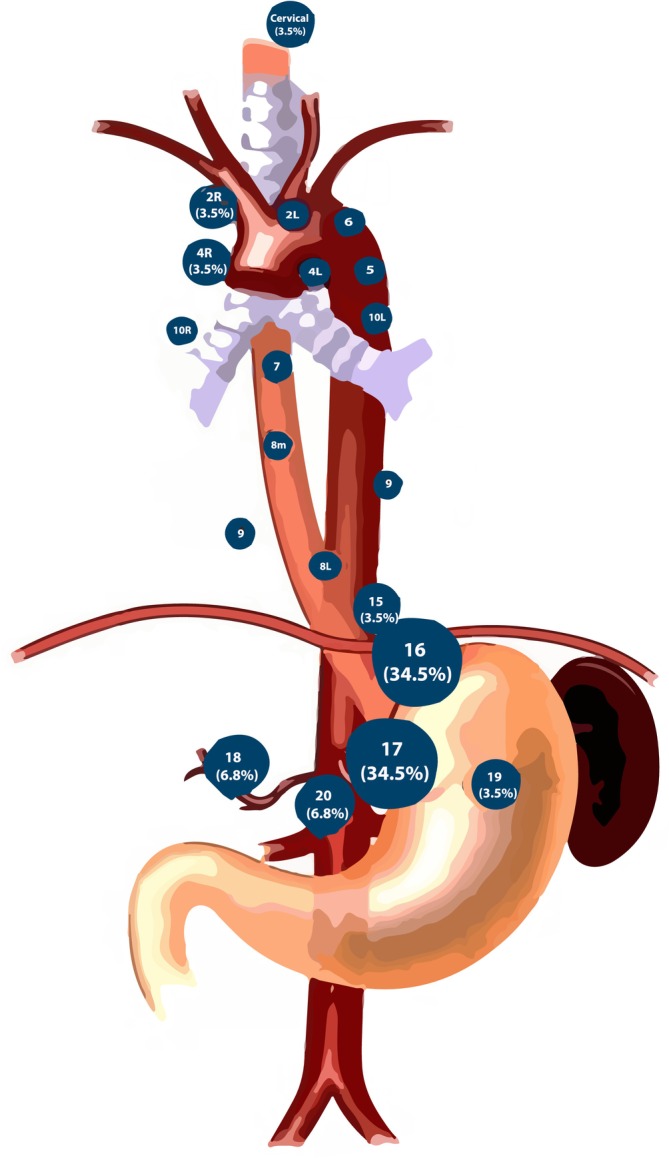
Out‐of‐field lymph nodes map.

Among the 306 patients, 29 (9.5% of the total patient population) experienced out‐of‐field LNs, 9 individuals from the IFI group and 20 from the ENI group (*p* = 0.724). Sex, age, BMI, KPS, smoking, drinking, location, clinical stage, and pre‐treatment with PET‐CT did not differ significantly between the out‐of‐field LN groups and the in‐field LNs group (*p* > 0.05). However, ECOG differed significantly in our study (*p* < 0.05). The information is shown in Table S1. Among patients with out‐of‐field LNs, 26 (93.1%) were in the abdominal LNs, whereas only 3 cases (6.9%) with out‐of‐field LNs were in the upper esophagus (Figure 3). According to the Kaplan–Meier curves (Figure S2), while there was no significant distinction in OS (S2A) and PFS (S2B) (OS, *p* = 0.2, HR = 0.59, 95% CI 0.31–1.12; PFS, *p* = 0.14, HR = 1.05, 95% CI 0.38–1.15), most patients with out‐of‐field LNs had lower survival rates compared to those with in‐field LNs. The out‐of‐field LN information of each patient is shown in Table S2.

## 4 Discussion

Our study featured the largest sample size among existing studies involving patients with ESCC undergoing nCRT. Our findings showed no notable variation in survival duration between ENI and IFI. Notably, the IFI group exhibited reduced radiation toxicity and no notable increase in out‐of‐field LNs.

With the widespread implementation of neoadjuvant therapies, the survival rates of patients with esophageal cancer have improved significantly [20, 21, 22]. Concurrently, researchers have reported side effects resulting from neoadjuvant radiotherapy, including myocardial infarction, arrhythmia, radiation‐induced myocarditis, radiation pneumonitis and leukopenia [6, 23, 24], which may diminish patient quality of life and, in severe cases, even lead to patient mortality. Therefore, controlling the side effects of radiation therapy and preventing missed irradiation are crucial.

nCRTs are currently the main treatment modalities for patients with locally advanced stage III–IVA ESCC. Nevertheless, researchers have focused on the irradiation range of the LN drainage areas during radical radiotherapy. In their prospective cohort study, Jing et al. reported that IFI provided similar OS and PFS with smaller volumes and lower toxicity than ENI. This is particularly beneficial in older patients as a smaller CTV can help reduce treatment‐related toxicities [25]. Yamashita et al. found that missed irradiation rates in clinically uninvolved nodal stations did not increase with IFI compared to ENI. Moreover, IFI resulted in significantly decreased esophageal toxicity [26]. However, research on lymphatic drainage areas in the context of neoadjuvant radiotherapy is limited, and large‐scale cohort studies are lacking. Moreover, with advancements in radiotherapy techniques, the complications and side effects induced by radiotherapy are also changing.

Our preliminary results demonstrated that the survival outcomes did not differ significantly, consistent with the results of other studies [27]. Before 2021, our center mainly adopted ENI irradiation (168/208 patients, 80.0%). However, after 2021, with the advancement of lymphatic drainage area‐related studies and recommendations from Chinese experts, IFI irradiation has gradually been adopted (64/98 patients, 65.3%). Substantial evidence has shown that the conventional approach of expanding the irradiation field to prolong survival is unnecessary. Post‐neoadjuvant treatment frequently involves comprehensive three‐field dissections that effectively clear suspicious positive LNs. Neoadjuvant chemotherapy may also play an important role.

We also observed a significantly lower incidence of bone marrow suppression and Grade ≥ 2 treatment‐related esophagitis in the IFI group than in the ENI group. In our study, compared to ENI, IFI significantly reduced the CTV and mean dose to the lungs, as well as the lung V_20_, V_30_, and V_40_ (*p* < 0.05). Volume reduction leads to decreased doses to critical organs, offering the potential for reduced symptoms such as radiation‐induced myocarditis and pneumonitis in the lungs. Moreover, reduced doses to the spinal cord may result in decreased hematological toxicity. IFI may improve patient tolerance and the quality of life during treatment. This reduced toxicity provides a foundation for expeditious surgical intervention after neoadjuvant therapy, thereby minimizing surgical complications. Our findings in the IFI group indicate that patients aged over 70 years developed more severe radiation esophagitis than their younger counterparts. This underscores the need to strengthen preventive measures and systematic assessment of radiotherapy‐related toxicities in the geriatric population (Table S6).

Meanwhile, in our center, the rate of out‐of‐field LN irradiation for patients with ESCC undergoing nCRT was 9.5%, whereas the missed irradiation rate in radical radiotherapy was 30.0% [28]. This may be related to the advancement of radiotherapy technology in recent years and the increased precision of postoperative pathology, and the missed irradiation rate is highest in abdominal LNs. Our study shows that the occurrence of out‐of‐field LN irradiation was not significantly associated with the LN irradiation method (*p* = 0.724), nor was it a significant factor for OS or PFS. Therefore, we should use a combination of various radiological examinations and pay attention to the abdominal LNs to clinically reduce the missed irradiation rate. Nonetheless, this research has some limitations: (1) it is a retrospective study and (2) patients underwent examinations or treatment at external institutions, which may have led to potential inaccuracies in the statistics of recurrence patterns and side effects. Moreover, while the reduction in treatment‐related toxicities is promising, the impact on long‐term survival outcomes is yet to be established. Further research, including larger‐scale prospective studies with longer follow‐up periods, is warranted to comprehensively evaluate the efficacy and safety of IFI in the treatment of ESCC.

## 5 Conclusion

Our findings support the use of IFI as a potentially advantageous treatment strategy for reducing acute treatment‐related esophagitis and pneumonitis in patients with ESCC treated with nCRT. Meanwhile, we have also demonstrated that IFI does not result in higher rates of out‐of‐field LN irradiation. The missed irradiation rate was highest in abdominal LNs. However, the clinical significance of these findings and their effect on long‐term survival outcomes requires further investigation.

## Author Contributions

**Xiaoding Zhou:** conceptualization; data curation; investigation; validation; writing – original draft; writing – review and editing; software. **Ying Liu:** investigation; project administration. **Jie Zhu:** methodology; formal analysis. **Jingqiu Li:** project administration; investigation. **Yi Wang:** supervision; methodology; software. **Guiyu Huang:** methodology; formal analysis; supervision. **Lin Peng:** project administration; data curation; writing – review and editing. **Yongtao Han:** data curation; writing – review and editing; supervision. **Xuefeng Leng:** data curation; supervision. **Chenghao Wang:** methodology; formal analysis. **Wenwu He:** methodology; formal analysis. **Lei Wu:** writing – review and editing; data curation; formal analysis; methodology. **Qifeng Wang:** funding acquisition; visualization; project administration; supervision; resources; data curation; methodology.

## Ethics Statement

This retrospective study was performed in line with the Declaration of Helsinki and approved by the Ethics Committee of Sichuan Cancer Hospital, thus alleviating patients of the requirement to sign a written informed consent form (ethics number, SCCHEC‐02‐2023‐029). All research experiments were conducted in accordance with the principles of the Declaration.

## Consent

The authors have nothing to report.

## Conflicts of Interest

The authors declare no conflicts of interest.

## Supporting information


**Table S1:** Comparison of patient characteristics between out‐of‐field LN and in‐field LN patients. BMI, body mass index; ECOG, Eastern Cooperative Oncology Group; ENI, elective lymph node irradiation; IFI, involved‐field irradiation; KPS, Karnofsky performance status; LN, lymph node; PET‐CT, positron emission tomography‐computed tomography.

**Table S2:** Detailed list of lymph node locations for each patient with missed irradiation. ENI, elective lymph node irradiation; IFI, involved‐field irradiation.

**Table S3:** Multivariate analysis of radiation pneumonitis for overall survival after neoadjuvant therapy using Cox proportional hazards model.

**Table S4:** Multivariate analysis of leukopenia for overall survival after neoadjuvant therapy using Cox proportional hazards model.

**Table S5:** Multivariate analysis of OS.

**Table S6:** Subgroup analyses of aged ≥ 70 years and aged < 70 years in two group in radiation esophagitis.

**Figure S1:** Patient inclusion and exclusion criteria.

**Figure S2:** Overall survival (A) and progression‐free survival (B) for patients in the out‐of‐field LN group and in‐field LN group. LN, lymph node.

## Data Availability

The data that support the findings of this study are available on request from the corresponding author. The data are not publicly available due to privacy or ethical restrictions.
