# Supplementary material for: Involved‐Field Irradiation Versus Elective Nodal Irradiation in Patients With Locally Advanced Esophageal Squamous Cell Carcinoma Treated With Neoadjuvant Chemoradiotherapy
Source: Cancer Med. 2025 Nov 30;14(23):e71392. doi: 10.1002/cam4.71392 (PMC12665187; doi:10.1002/cam4.71392)
Supplement: Supplementary file 1 — Table S1: Comparison of patient characteristics between out‐of‐field LN and in‐field LN patients. BMI, body mass index; ECOG, Eastern Cooperative Oncology Group; ENI, elective lymph node irradiation; IFI, involved‐field irradiation; KPS, Karnofsky performance status; LN, lymph node; PET‐CT, positron emission tomography‐computed tomography. [file CAM4-14-e71392-s005.docx]

**Supplement Table 1: Comparison of patient characteristics between out-of-field LN and in-field LN patients.**

| Variables | Out-of-field LNs  (n = 29) | In-field LNs  (n = 277) | *p* |
| --- | --- | --- | --- |
| Age |  |  | 0.843 |
| < 65 | 20 (69.0) | 186 (67.1) |  |
| ≥ 65 | 9 (31.0) | 91 (32.9) |  |
| Sex |  |  | 1 |
| Male | 24 (82.8) | 231 (83.4) |  |
| Female | 5 (17.2) | 46 (16.6) |  |
| Smoking |  |  | 0.471 |
| No | 12 (41.4) | 96 (34.7) |  |
| Yes | 17 (58.6) | 181 (65.3) |  |
| Drinking |  |  | 0.923 |
| No | 10 (34.5) | 98 (35.4) |  |
| Yes | 19 (65.5) | 179 (64.6) |  |
| BMI |  |  | 0.703 |
| < 18.5 | 3 (10.3) | 19 (6.9) |  |
| 18.5-24 | 17 (58.6) | 167 (60.3) |  |
| > 24 | 9 (31.0) | 91 (32.9) |  |
| ECOG |  |  | 0.029 |
| 0 | 19 (65.5) | 228 (82.3) |  |
| 1 | 10 (34.5) | 49 (17.7) |  |
| KPS |  |  | 0.780 |
| 70-80 | 3 (10.3) | 41 (14.8) |  |
| 90-100 | 26 (89.7) | 236 (85.2) |  |
| Location |  |  | 0.168 |
| Upside | 4 (13.8) | 36 (13) |  |
| Middle | 8 (27.6) | 121 (43.7) |  |
| Lower | 17 (58.1) | 120 (43.3) |  |
| Clinical stage |  |  | 0.155 |
| II | 4 (13.4) | 14 (5.1) |  |
| III | 19 (65.5) | 202 (72.9) |  |
| IV | 6 (20.7) | 61 (22) |  |
| LN irradiation |  |  | 0.724 |
| ENI | 20 (69.0) | 182 (65.7) |  |
| IFI | 9 (31.0) | 95 (34.3) |  |
| Pre-treatment PET-CT |  |  | 0.108 |
| No | 25 (86.2) | 261 (94.2) |  |
| Yes | 4 (13.8) | 16 (5.8) |  |

ENI, elective lymph node irradiation; IFI, involved field irradiation; BMI, Body Mass Index; ECOG, Eastern Cooperative Oncology Group; KPS, Karnofsky Performance Status; PET-CT, positron emission tomography-computed tomography; LN, lymph node.
