# Supplementary material for: Involved‐Field Irradiation Versus Elective Nodal Irradiation in Patients With Locally Advanced Esophageal Squamous Cell Carcinoma Treated With Neoadjuvant Chemoradiotherapy
Source: Cancer Med. 2025 Nov 30;14(23):e71392. doi: 10.1002/cam4.71392 (PMC12665187; doi:10.1002/cam4.71392)
Supplement: Supplementary file 2 — Table S2: Detailed list of lymph node locations for each patient with missed irradiation. ENI, elective lymph node irradiation; IFI, involved‐field irradiation. [file CAM4-14-e71392-s004.docx]

**Supplement Table 2: Detailed list of lymph node locations for each patient with missed irradiation**

| Patient ID | LNs irradiation | The out-of-field  LN location | The out-of-field  LN grouping | Pre-treatment PET-CT |
| --- | --- | --- | --- | --- |
| 1 | ENI | abdomen | 16 | No |
| 2 | ENI | cervical | left cervical | No |
| 3 | ENI | abdomen | 16 | No |
| 4 | ENI | abdomen | 17 | No |
| 5 | ENI | abdomen | 16 | No |
| 6 | ENI | abdomen | 17 | Yes |
| 7 | IFI | abdomen | 17 | No |
| 8 | ENI | abdomen | 17 | Yes |
| 9 | ENI | abdomen | 16 | No |
| 10 | ENI | abdomen | 15 | No |
| 11 | ENI | abdomen | 20 | No |
| 12 | ENI | abdomen | 17 | No |
| 13 | ENI | abdomen | 20 | No |
| 14 | ENI | abdomen | 17 | No |
| 15 | ENI | abdomen | 16 | No |
| 16 | ENI | abdomen | 16 | No |
| 17 | ENI | abdomen | 17 | No |
| 18 | IFI | abdomen | 16 | No |
| 19 | IFI | abdomen | 17 | No |
| 20 | ENI | abdomen | 16 | No |
| 21 | ENI | abdomen | 16 | Yes |
| 22 | ENI | abdomen | 18 | No |
| 23 | IFI | upper | 2r | Yes |
| 24 | ENI | abdomen | 19 | No |
| 25 | IFI | abdomen | 17 | No |
| 26 | IFI | upper | 4r | No |
| 27 | IFI | abdomen | 17 | No |
| 28 | IFI | abdomen | 16 | No |
| 29 | IFI | abdomen | 18 | No |

ENI, elective lymph node irradiation; IFI, involved field irradiation;
