# Supplementary material for: Involved‐Field Irradiation Versus Elective Nodal Irradiation in Patients With Locally Advanced Esophageal Squamous Cell Carcinoma Treated With Neoadjuvant Chemoradiotherapy
Source: Cancer Med. 2025 Nov 30;14(23):e71392. doi: 10.1002/cam4.71392 (PMC12665187; doi:10.1002/cam4.71392)
Supplement: Supplementary file 3 — Table S3: Multivariate analysis of radiation pneumonitis for overall survival after neoadjuvant therapy using Cox proportional hazards model. [file CAM4-14-e71392-s001.docx]

**Supplement table 3: multivariate analysis of radiation pneumonitis for overall survival after neoadjuvant therapy using Cox proportional hazards model**

| Variables | HR (95% CI) | *p* |
| --- | --- | --- |
| Smoking |  |  |
| No | 1 |  |
| Yes | 1.15 (0.69~1.93) | 0.59 |
| Drinking |  |  |
| No | 1 |  |
| Yes | 0.96 (0.59~1.56) | 0.87 |
| Location |  |  |
| Upside | 1 |  |
| Middle | 1.71 (0.76~3.85) | 0.197 |
| Lower | 1.27 (0.56~2.87) | 0.566 |
| Clinical stage |  |  |
| II | 1 |  |
| III | 1.03 (0.32~3.33) | 0.961 |
| IVA | 1.81 (0.55~5.97) | 0.332 |
