# Supplementary material for: Involved‐Field Irradiation Versus Elective Nodal Irradiation in Patients With Locally Advanced Esophageal Squamous Cell Carcinoma Treated With Neoadjuvant Chemoradiotherapy
Source: Cancer Med. 2025 Nov 30;14(23):e71392. doi: 10.1002/cam4.71392 (PMC12665187; doi:10.1002/cam4.71392)
Supplement: Supplementary file 4 — Table S4: Multivariate analysis of leukopenia for overall survival after neoadjuvant therapy using Cox proportional hazards model. [file CAM4-14-e71392-s003.docx]

**Supplement table 4: multivariate analysis of leukopenia for overall survival after neoadjuvant therapy using Cox proportional hazards model**

| Variables | HR (95% CI) | *p* |
| --- | --- | --- |
| Smoking |  |  |
| No | 1 |  |
| Yes | 0.82 (0.62~1.08) | 0.162 |
| Drinking |  |  |
| No | 1 |  |
| Yes | 0.87 (0.66~1.15) | 0.335 |
| Location |  |  |
| Upside | 1 |  |
| Middle | 0.95 (0.63~1.44) | 0.815 |
| Lower | 0.88 (0.59~1.31) | 0.527 |
| Clinical stage |  |  |
| II | 1 |  |
| III | 1.15 (0.6~2.19) | 0.668 |
| IV | 1.02 (0.52~2.02) | 0.951 |
