# Supplementary material for: Involved‐Field Irradiation Versus Elective Nodal Irradiation in Patients With Locally Advanced Esophageal Squamous Cell Carcinoma Treated With Neoadjuvant Chemoradiotherapy
Source: Cancer Med. 2025 Nov 30;14(23):e71392. doi: 10.1002/cam4.71392 (PMC12665187; doi:10.1002/cam4.71392)
Supplement: Supplementary file 5 — Table S5: Multivariate analysis of OS. [file CAM4-14-e71392-s008.docx]

**Supplement table 5: multivariate analysis of OS**

| Variables | HR (95% CI) | *p* |
| --- | --- | --- |
| Smoking |  |  |
| No | 1 |  |
| Yes | 1.34 (0.83~2.15) | 0.231 |
| Drinking |  |  |
| No | 1 |  |
| Yes | 1.16 (0.73~1.83) | 0.536 |
| Location |  |  |
| Upside | 1 |  |
| Middle | 1.36 (0.68~2.73) | 0.388 |
| Lower | 1.05 (0.52~2.11) | 0.884 |
| Clinical stage |  |  |
| II | 1 |  |
| III | 1.05 (0.33~3.36) | 0.935 |
| IV | 1.79 (0.55~5.86) | 0.338 |
