# Supplementary material for: Involved‐Field Irradiation Versus Elective Nodal Irradiation in Patients With Locally Advanced Esophageal Squamous Cell Carcinoma Treated With Neoadjuvant Chemoradiotherapy
Source: Cancer Med. 2025 Nov 30;14(23):e71392. doi: 10.1002/cam4.71392 (PMC12665187; doi:10.1002/cam4.71392)
Supplement: Supplementary file 6 — Table S6: Subgroup analyses of aged ≥ 70 years and aged < 70 years in two group in radiation esophagitis. [file CAM4-14-e71392-s007.docx]

Supplement table 6: Subgroup analyses of aged ≥70 years and aged < 70 years in two group in radiation esophagitis.

| Subgroup | Variables | n | n. event (%) | HR (95% CI) | crude.P_value | p |
| --- | --- | --- | --- | --- | --- | --- |
| ENI or IFI |  |  |  |  |  | 0.074 |
| ENI | Age < 70 | 186 | 60 (32.3) | 1(Ref) |  |  |
|  | Age ≥ 70 | 16 | 4 (25) | 0.86 (0.31~2.37) | 0.3 |  |
| IFI | Age < 70 | 93 | 21 (22.6) | 1(Ref) |  |  |
|  | Age ≥ 70 | 11 | 3 (27.3) | 1.56 (0.46~5.26) | 0.005 |  |
