# Supplementary material for: Involved‐Field Irradiation Versus Elective Nodal Irradiation in Patients With Locally Advanced Esophageal Squamous Cell Carcinoma Treated With Neoadjuvant Chemoradiotherapy
Source: Cancer Med. 2025 Nov 30;14(23):e71392. doi: 10.1002/cam4.71392 (PMC12665187; doi:10.1002/cam4.71392)
Supplement: Supplementary file 7 — Figure S1: Patient inclusion and exclusion criteria. [file CAM4-14-e71392-s002.docx]

**Supplementary Appendix Figure 1:** Patient inclusion and exclusion criteria.
