# Supplementary material for: Involved‐Field Irradiation Versus Elective Nodal Irradiation in Patients With Locally Advanced Esophageal Squamous Cell Carcinoma Treated With Neoadjuvant Chemoradiotherapy
Source: Cancer Med. 2025 Nov 30;14(23):e71392. doi: 10.1002/cam4.71392 (PMC12665187; doi:10.1002/cam4.71392)
Supplement: Supplementary file 8 — Figure S2: Overall survival (A) and progression‐free survival (B) for patients in the out‐of‐field LN group and in‐field LN group. LN, lymph node. [file CAM4-14-e71392-s006.docx]

**Supplementary Appendix Figure 2:** Overall survival (A) and progression-free survival (B) for patients in the out-of-field LN group and in-field LN group. LN, lymph node.
